# T-cell receptor dynamics in digestive system cancers: a multi-layer machine learning approach for tumor diagnosis and staging

**DOI:** 10.3389/fimmu.2025.1556165

**Published:** 2025-04-08

**Authors:** Changjin Yuan, Bin Wang, Hong Wang, Fang Wang, Xiangze Li, Ya’nan Zhen

**Affiliations:** ^1^ Clinical Laboratory, Affiliated Hospital of Shandong University of Traditional Chinese Medicine, Jinan, China; ^2^ Minimally Invasive Surgery, The Third Affiliated Hospital of Shandong First Medical University, Jinan, China; ^3^ Department of Gastrointestinal Surgery, Shandong Provincial Third Hospital, Shandong University, Jinan, China; ^4^ Department of Gastrointestinal Surgery, The Third Affiliated Hospital of Shandong First Medical University, Jinan, China

**Keywords:** T-cell receptor repertoire (TCR), colorectal cancer (CRC), gastric cancer (GC), multi-layer machine learning, diagnostic model

## Abstract

**Background:**

T-cell receptor (TCR) repertoires provide insights into tumor immunology, yet their variations across digestive system cancers are not well understood. Characterizing TCR differences between colorectal cancer (CRC) and gastric cancer (GC), as well as developing machine learning models to distinguish cancer types, metastatic status, and disease stages are crucial for guiding clinical practices.

**Methods:**

A cohort study of 143 tumor patients (96 CRC, 47 GC) was conducted. High-throughput TCR sequencing was performed to capture TCR beta (TRB), delta (TRD), and gamma (TRG) chain data. Tissue-specific patterns in TCR repertoire features, such as V-J gene recombination, complementarity-determining region 3 (CDR3) sequences, and motif distributions, were analyzed. Multi-layer machine learning-based diagnostic models were developed by leveraging motif-based feature and deep learning-based feature extraction using ProteinBERT from the 100 most abundant CDR3 sequences per sample. These models were used to differentiate CRC from GC, distinguish between primary and metastatic CRC lesions, and predict disease stages in CRC.

**Results:**

Tissue-specific differences in TCR repertoires were observed across CRC, GC, and between primary and metastatic lesions, as well as across disease stages in CRC. Distinct V-J gene recombination patterns were identified, with CRC showing enrichment in TRBV*-TRBJ* combinations, while GC exhibited higher levels of γδT-cell-related recombination. Primary and metastatic lesions of CRC patients displayed distinct V-J recombination preferences (e.g., *TRBV7-9*/*TRBJ2-1* higher in metastatic; *TRBV20-1*/*TRBJ1-2* higher in primary) and CDR3 sequence differences, with metastatic having shorter TRG CDR3 lengths (*p*-value = 0.019). Across CRC stages, later stages (III–IV) showed higher clonal diversity (*p*-value < 0.05) and stage-specific V-J patterns, alongside distinct CDR3 amino acid preferences at N-terminal (positions 1–2) and central positions (positions 5–12). Multi-dimensional machine learning models demonstrated exceptional diagnostic performance across all classification tasks. For distinguishing CRC from GC, the model achieved an accuracy of 97.9% and an area under the curve (AUC) of 0.996. For differentiating primary from metastatic CRC, the model achieved 100% accuracy with an AUC of 1.000. In predicting CRC disease stages, the model attained an accuracy of 96.9% and an AUC of 0.993. Extensive validation using simulated and publicly available datasets, confirmed the robustness and reliability of the models, demonstrating consistent performance across diverse datasets and experimental conditions.

**Conclusions:**

Our investigation provides novel insights into TCR repertoire variations in digestive system tumors, and highlight the potential of immune repertoire features as powerful diagnostic tools for understanding cancer progression and potentially improving clinical decision-making.

## 1 Introduction

The immune system plays a crucial role in cancer defense, with T cells being key mediators of immune responses. T-cell receptors (TCRs) are responsible for recognizing tumor-associated antigens presented by major histocompatibility complex (MHC) molecules on the surface of tumor cells (1–3). The diversity of TCRs enables the immune system to recognize a broad spectrum of tumor antigens, making TCR analysis a critical area in cancer immunology research (3). However, a thorough understanding of TCR repertoire differences, especially in gastrointestinal cancers, remains limited (4, 5).

In recent years, molecular diagnostic techniques, such as DNA methylation and mutation analysis, have significantly advanced early cancer detection and patient stratification (6–8). Notably, the analysis of circulating free DNA (cfDNA) combined with machine learning has proven effective in early detection of cancers such as esophageal squamous cell carcinoma (9, 10). When integrated with TCR repertoire analysis, these technologies provide a comprehensive view of tumor immune responses and offer potential biomarkers for early detection and targeted therapy.

Innovations in high-throughput and single-cell sequencing technologies have enabled detailed characterization of TCR repertoires (11). Tools such as multiplexed pMHC multimers and TCRconv (12) aid in identifying T cells specific to certain antigens and predicting the interactions between TCRs and antigen epitopes, thereby enhancing our understanding of tumor immunology and providing crucial insights for immunotherapy strategies (12–14). Furthermore, deep learning frameworks like DeepTCR (15) have been employed to analyze complex TCR data, helping to deepen our understanding of immune responses and enhance predictions of responses to immunotherapies, such as immune checkpoint inhibitors and CAR-T cell therapies. Similarly, DeepCAT (16) and DeepLION (17) provide CNN-based models for predicting patient statuses using TCR CDR3 sequences. Research on public TCRs—sequences shared across individuals—has furthered our understanding of tumor immune responses. For example, conserved complementarity-determining region 3 (CDR3) motifs identified in breast cancer suggest that these shared sequences may serve as universal biomarkers for immunotherapy (18, 19). Additionally, tissue-specific variations in TCR repertoires across different tumor types (including V-J gene rearrangement patterns, which reflect the composition of post-selection TCR sequences) provide insights into how tumors evade immune surveillance and may guide strategies to enhance anti-tumor immunity (19).

Colorectal cancer (CRC) and gastric cancer (GC) are two common malignancies of the digestive system with distinct immune features. CRC, due to the high heterogeneity of its tumor microenvironment, employs various immune escape mechanisms to promote tumor progression (20–22). In contrast, GC is often associated with chronic inflammation and Helicobacter pylori infection, complicating its immune response due to altered inflammatory mechanisms (23–25). Despite these distinct immune backgrounds, systematic analyses of TCR repertoires in CRC and GC remain scarce, limiting our understanding of their immune characteristics.

The present study investigates the differential TCR repertoires in gastrointestinal cancers through high-throughput sequencing and advanced machine learning methodologies. It focuses on critical features such as V-J gene rearrangements and CDR3 sequence motifs to identify immune signatures specific to these malignancies. Leveraging CDR3 sequences and motif characteristics, we develop multi-layered clinical diagnostic prediction models tailored to diverse applications, which are rigorously evaluated using independent internal, simulated, and external test datasets, demonstrating robust performance. By integrating molecular diagnostics with computational strategies, our findings refine diagnostic accuracy and advance the understanding of cancer immunology.

## 2 Materials and methods

### 2.1 Study participants

From 2018 to 2024, 143 fresh tumor samples were collected from patients who underwent surgical resection at the Third Affiliated Hospital of Shandong First Medical University (Affiliated Hospital of Shandong Academy of Medical Sciences). The inclusion criteria were: (1) histopathological confirmation of primary colon, liver, or gastric cancer, and (2) availability of complete clinical data. Patients were excluded if they met any of the following criteria: (1) prior treatment with radiotherapy, chemotherapy, immunotherapy, or targeted therapy before surgery, (2) a history of other malignancies, or (3) the presence of autoimmune diseases or chronic conditions (see **Supplementary Table S1** for details).

This study was approved by the Ethics Committee of the Third Affiliated Hospital of Shandong First Medical University (Approval No. FY2021018). Written informed consent was obtained from all participants prior to enrollment. Tumor specimens were collected immediately after surgical excision, washed with ice-cold saline, and promptly cryopreserved in liquid nitrogen for further analysis.

### 2.2 Tumor tissue RNA extraction and bulk T-cell receptor sequencing

Tumor tissue samples (≥ 2 mL) were collected in EDTA vacutainer tubes, and total RNA was extracted using the RNAsimple Total RNA Kit (DP419, Tiangen Biotech, Beijing, China). RNA concentrations were measured with a NanoDrop ND-2000 spectrophotometer (Thermo Scientific, UK). cDNA synthesis and multiplex PCR amplification of rearranged TCR *α*, *β*, *δ*, *γ*-chains sequences were performed using Immune Repertoire Library Preparation Kits (Geneway, Jinan, China) following a previously described protocol (26, 27). TCR libraries were sequenced on a DNBSEQ-T7 platform (MGI, Shenzhen, China), generating paired-end 150 bp reads.

### 2.3 Preprocessing of sequencing data using MiXCR tool

Sequencing data were stored in FASTQ format, with raw reads demultiplexed according to index primer sequences specific to each sample. Low-quality sequences were removed during quality control, and the remaining reads were mapped to the V, D, and J gene segments of TCR *α, β, δ, γ* chains using MiXCR version 4.3.2 (28), employing default parameters for alignment and clonotype assembly. TCR reference gene data were sourced from the IMGT database (http://www.imgt.org/vquest/refseqh.html).

### 2.4 Diversity metrics of T-cell receptors

To assess immune repertoire diversity, we computed Shannon diversity, Simpson diversity, richness, evenness, top clone fraction, and the number of clones contributing to 50% of the repertoire. Shannon diversity was calculated as $-\sum p_{i}\log p_{i}$ , where $p_{i}$ represents the proportion of each clone. Simpson diversity was estimated as $1-\sum p_{i}\log p_{i}$ . Richness was defined as the total number of unique clones, and evenness was derived as the ratio of Shannon diversity to the logarithm of richness. The top clone fraction was determined as the maximum clone proportion, and the number of clones constituting 50% of the total repertoire was obtained by summing the largest clone proportions until the cumulative sum exceeded 0.5. Diversity metrics were computed for each sample and integrated with corresponding metadata for further analysis.

### 2.5 V-J gene preferences analysis across different groups

V-J gene preferences were analyzed by calculating the frequency of each V-J pair within each group, and normalizing these frequencies by the total count of V-J pairs in that group. A matrix of V-J pair frequencies was constructed, with rows representing the V-J pairs and columns corresponding to the different groups. The differences in V-J gene usage between groups were assessed by computing the log-transformed ratio (log2) of the frequencies between groups. This analysis was extended to include various cancer types, as well as the PT and MT subgroups of CRC, and the TNM stages of CRC. The resulting differences in V-J gene usage were visualized through a heatmap, which employed a color gradient to display the magnitude of these differences, highlighting variations in V-J gene preferences across the groups.

### 2.6 Determination of the specific motifs between different groups

A “Seurat” object was created using *CreateSeuratObject* with the *k*-mer count data (*k* = 5) (29). The data was then normalized using *NormalizeData*. The type of each sample was assigned as the identity class. To identify specific motifs associated with each cancer type, the *FindAllMarkers* function was applied. The specific motifs were filtered by a *p*-value threshold of 0.01 (*return.thresh* = 0.01).

### 2.7 Multi-layer machine learning for distinguishing cancer types and staging

To distinguish between CRC and GC, primary tumors (PT) vs. metastatic tumors (MT) within CRC, and various TNM stages of CRC, a machine learning framework integrates sequence-based features derived from CDR3 sequences using ProteinBERT (30) and motif-based features extracted from CDR3 sequences with the “immunarch” package (version 1.0.0) (31). ProteinBERT encodes CDR3 sequence data by selecting the 100 most abundant sequences for each sample, followed by principal component analysis (PCA) for dimensionality reduction, retaining the top 50 principal components. Motif features, are generated by extracting 5-mers from the CDR3 sequence data, which are also reduced by PCA method. Both PCA-reduced CDR3 features and motif features are then used as inputs for model training.

In the first layer of the model, base classifiers—including Generalized Linear Models (GLM), XGBoost, Random Forest, and Neural Networks—are trained on the combined feature set. For GLM, the *alpha* parameter, which controls the strength of regularization, is tuned across values of 0, 0.2, 0.4, 0.6, 0.8, and 1. This parameter influences the trade-off between *L1* (*lasso*) and *L2* (*ridge*) regularization. For XGBoost, hyperparameters such as the number of boosting rounds (*nrounds*), set to 100 or 200, the maximum tree depth (*max_depth*), adjusted to 3 or 6, the learning rate (*eta*), tested at 0.01 or 0.1, the gamma parameter, tested at 0 and 0.1, and the subsample ratio (*subsample*), tested at 0.7, 0.8, or 0.9, are optimized. Random Forest models are tuned with respect to the number of features considered for splitting (*mtry*), set to 2, 4, or 6, and the number of trees (*ntree*), set to 500 or 1000. For Neural Networks, hyperparameters such as the number of neurons in the hidden layer (*size*), set to 3, 4, or 5, and the weight decay (*decay*), set to 0.001, 0.01, or 0.1, are optimized. After training, the top-performing models for each feature type (ProteinBERT-derived features and motif features) are ranked by their AUC (Area Under the Curve) scores. The five best models for each feature set are then selected and used to generate predictions for each sample (a total of 40 models).

In the second layer, stacking models—including GLM, XGBoost, Random Forest, and Neural Networks—are trained using the predictions from the first layer as input. These stacking models are similarly optimized, with GLM tuning the alpha parameter, XGBoost adjusting the number of boosting rounds (*nrounds*), maximum tree depth (*max_depth*), and learning rate (*eta*), Random Forest fine-tuning the number of trees (*ntree*), number of features considered for each split (*mtry*), and Neural Networks setting neurons in the hidden layer (*size*). The top ten models from the second layer are selected based on their AUC scores, and the final ensemble model is obtained by combining the predictions (averaging for prediction scores) from the second layer. This ensemble approach enhances classification performance and improves the model’s ability to distinguish between groups.

### 2.8 Evaluation of model performance using AUC, accuracy, sensitivity, and specificity

To evaluate the performance of the models, we calculated key metrics including Area Under the Curve (AUC), Accuracy, Sensitivity, and Specificity. The AUC was determined by generating a Receiver Operating Characteristic (ROC) curve using the roc function from the “pROC” package (version 1.18.5) (32), comparing the predicted scores with the true labels. The resulting True Positive Rate (TPR) and False Positive Rate (FPR) were plotted using “ggplot2” (version 3.5.1) (33), with a dashed diagonal line representing thresholds for classification. Accuracy, Sensitivity (TPR), and Specificity (True Negative Rate) were derived from the *confusionMatrix* function from “caret” package (version 6.0-94) (34) for each model, providing a comprehensive assessment of model performance.

### 2.9 Simulation of testing data for model performance evaluation

Extended testing data were simulated using TCR data from the output of the MiXCR tool (version 4.3.2) (28). The input dataset was down sampled based on the type attribute to generate test datasets for various groups (e.g., CRC, GC). For example, when simulating data for CRC, the dataset was filtered to include only entries where type == ‘CRC’, resulting in a positive subset. Within each subset, 50% of the unique clone identifiers were randomly selected. Clone fractions (*cloneFraction*) were normalized to sum to one, and simulated reads were generated by sampling clones with probabilities proportional to their clone fractions, ensuring that more abundant clones were sampled more frequently. The resulting dataset was grouped by the unique sequence identifier (*aaSeqCDR3*), the clone counts and adjusted *cloneFraction* were calculated for each sequence. The dataset was sorted by *cloneFraction* in descending order, and a new “cloneId” was assigned starting from zero. Each simulated dataset was saved as a TSV file, with filenames incorporating the label (e.g., ‘CRC’) and a unique simulation ID. For each group, 100 simulated datasets were generated, each containing 1,000,000 reads. The generated data were then fed into “immunarch” to obtain motif matrices and into ProteinBERT to extract CDR3 sequencing information.

### 2.10 Comparison with existing methods

To compare the performance of our method with other publicly available tools, we included DeepLION (17) and DeepCAT (16). DeepLION processes TCR sequences and extracts features using a convolutional neural network (CNN), with a single-layer linear transformation used as the classifier. DeepCAT first applies *iSMART* (18) to perform similarity clustering on the sequences from each sample, followed by the use of five CNN models to make predictions based on varying amino acid (AA) lengths (ranging from 12 to 16) (35).

To evaluate the performance of DeepLION and DeepCAT on the simulated TCR dataset (see Materials and Methods section 2.8), we used our real TCR dataset as the training set. Classification models were trained for the GC vs. CRC, PT vs. MT, and Earlier vs. Later categories, following the training procedures outlined in the manuals of both tools and using default parameters. The trained models were then applied to predict the labels for the corresponding simulated TCR datasets. For external validation, we used the Lung (n = 444) (36) and thyroid carcinoma (THCA) (n = 430) (37) datasets, which were randomly split into training and independent test sets at a 7:3 ratio. The multi-layer classification model (Ours), DeepLION, and DeepCAT were trained on the training set and evaluated on the test set.

### 2.11 Estimation of immune infiltration and tumor mutational burden

To estimate immune infiltration in the colorectal adenocarcinoma (COAD) data from “The Cancer Genome Atlas” (TCGA) (TCGA-COAD) (n = 521), the deconvolution tool CIBERSORT (38) was applied, utilizing the LM22 reference matrix provided by Newman et al. (38), and bulk expression profiles were extracted from the TCGA-COAD transcriptome data. Tumor mutational burden (TMB) was calculated using the *tmb* function from the “maftools” package (version 2.20.0) (39).

To evaluate potential confounding factors, such as immune infiltration and TMB, associated with CRC patient states, a multi-layer approach, as described in Section 2.6, was employed for model training based on the TCGA-COAD cohort. Immune infiltration and TMB were used separately as features in the model. The objective was to distinguish between normal and primary tumor tissues, as well as between tumor stages (Earlier, combining Stage I-II, and Later, combining stages ≥ III).

### 2.12 Statistical analysis

Student’s *t*-test and the Wilcoxon rank-sum test, were employed to compare statistical differences within the study. Principal component analysis (PCA) and *t*-Distributed Stochastic Neighbor Embedding (*t*-SNE) were employed for dimensionality reduction and visualization of the samples’ distribution, respectively. Specificity, sensitivity, accuracy, and the Area Under the Curve (AUC) were used to evaluate the performance of the classification model. All statistical analyses were executed using R version 4.3.2.

## 3 Results

### 3.1 Tissue-specific differences in TCR repertoire characteristics across digestive system tumors

**Figure 1:**
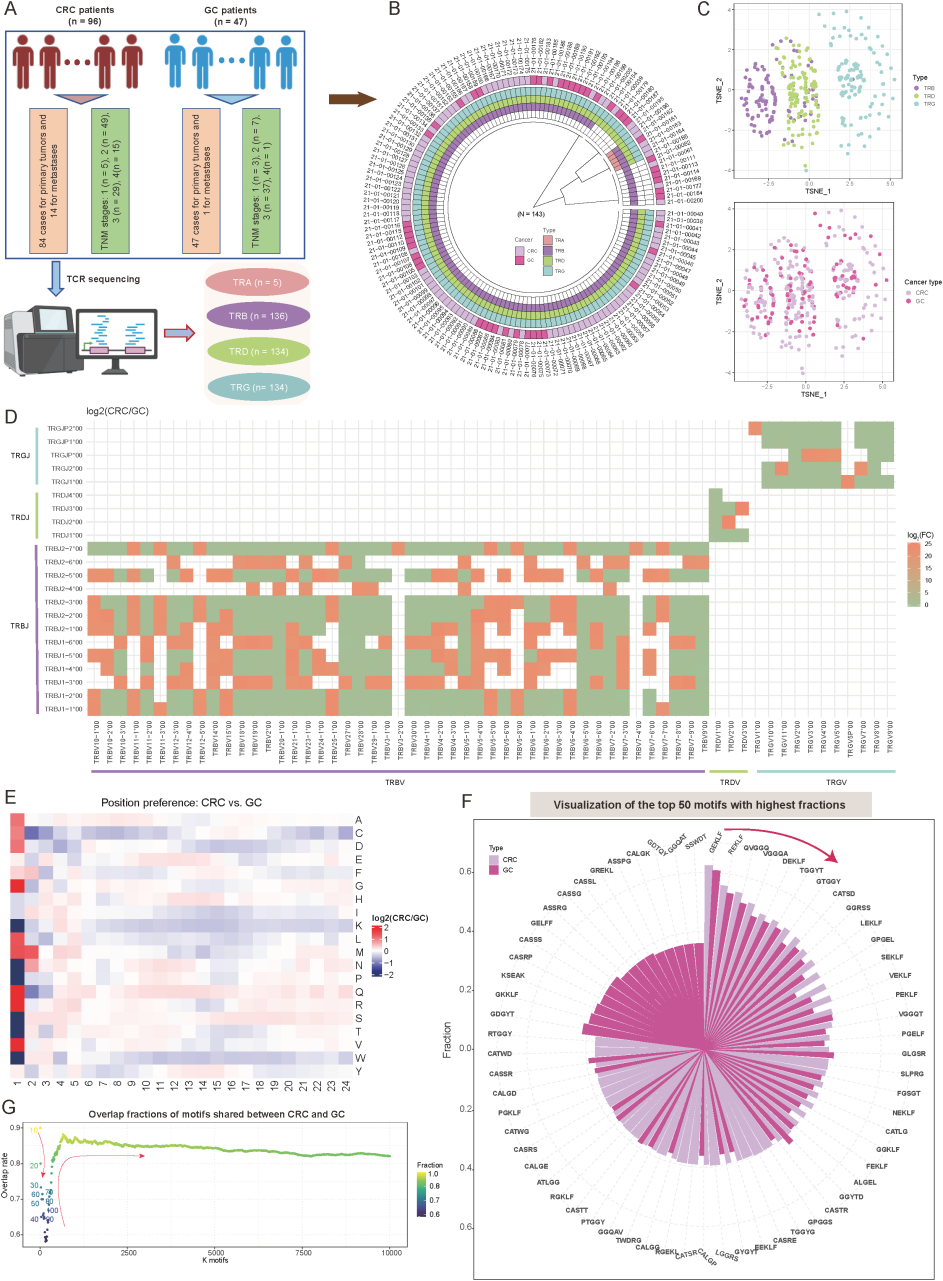
Overview of T-cell receptor (TCR) repertoire characteristics and preferences in colorectal cancer (CRC) and gastric cancer (GC) patients. **(A)** Overview of the study sample grouping, illustrating the classification framework and sample sources. **(B)** Circular diagram summarizing the tissue origins of cancer samples and the corresponding TCR receptor sequencing outcomes. **(C)** Scatter plots showing *t*-distributed stochastic neighbor embedding (*t*-SNE)-based distributions of TCR repertoires (top panel) and cancer types (bottom panel). The *t*-SNE plots were constructed based on repertoire overlap between samples, calculated using the “immunarch” package (version 1.0.0) (31). Each point represents an individual sample, with colors denoting distinct groups. **(D)** Heatmap comparing V-J combination preferences between CRC and GC patients. The color intensity represents the log2 ratio of specific V-J combination abundances in CRC relative to GC. Red indicates enrichment in CRC, while green indicates enrichment in GC. V-J combinations detected in fewer than 100 clones were excluded. **(E)** Heatmap showing the abundance preferences of 20 amino acids (AA) at various positions within CDR3 sequences in CRC and GC patients. The color intensity denotes the log2 ratio of amino acid abundances in CRC compared to GC. Analysis includes clones with CDR3 lengths between 5 and 24. **(F)** Circle bar plot showing the top 50 motifs with the highest fractions in CRC and GC patients. The y-axis represents the fraction of each motif, calculated as the count of a specific motif divided by the total motif counts for each cancer type. **(G)** Scatter plot depicting the distribution of motif overlap ratios between CRC and GC patients as a function of the number of selected motifs. Motifs are ranked by fraction in descending order for each cancer type.

To investigate the characteristic differences in TCR repertoires among tumors of the digestive system, we conducted a cohort study comprising 143 samples (**Figure 1A**, **Supplementary Table S1**). The colorectal cancer (CRC) cohort (n = 96) included 84 primary tumors and 14 metastatic lesions, distributed across tumor-node-metastasis (TNM) stages as follows: stage I (5.2%, n = 5), stage II (51.0%, n = 49), stage III (30.2%, n = 29), and stage IV (15.6%, n = 15) (**Figures 1A, B**, **Supplementary Table S1**). The gastric cancer (GC) cohort (n = 47) consisted of 46 primary tumors and one metastatic lesion, predominantly at stage III (78.7%, n = 37), followed by stage II (14.9%, n = 7). All samples underwent high-throughput bulk TCR sequencing, yielding comprehensive TCR beta (TRB) (n = 136), TCR delta (TRD) (n = 134), and TCR gamma (TRG) (n = 134) data (**Figures 1A, B**, **Supplementary Table S1**). TCR alpha (TRA) data were obtained for five samples, but these were excluded from subsequent analyses to avoid statistical bias due to the limited sample size. The comparison of TCR repertoires between CRC and GC tumors provides valuable insights into immune mechanisms specific to these tumor types within the digestive system and may inform therapeutic strategies. Therefore, we undertook the following comparative analysis.

Initially, dimensionality reduction via *t*-distributed stochastic neighbor embedding (*t*-SNE) based on immune repertoire overlap revealed distinct TCR chain-specific distribution patterns. Specifically, samples from the same TCR chain exhibited closely spatial clustering, while those from different chains displayed clear separation (**Figure 1C**). This distribution suggests unique functional roles for different TCR chains in immune recognition (40). Interestingly, when tumor type information was mapped onto the same dimensionality-reduced space, CRC and GC samples showed a relatively uniform distribution (**Figure 1C**). The diversity metrics, including Shannon diversity, Simpson diversity, evenness, and richness, showed no notable differences between CRC and GC (**Supplementary Figure S1A**; *p-value* < 0.05; see Materials and Methods). This seemingly contradictory phenomenon implies that intrinsic TCR chain characteristics may play a more dominant role in shaping immune repertoire features than tumor type. A systematic investigation of variable (V) and joining (J) gene recombination patterns identified significant, tissue-specific preferences between CRC and GC (see Materials and Methods). For the *β* chain, several TCR variable beta (*TRBV*)–TCR joining beta (*TRBJ*) combinations, such as TRBV10-1*00/TRBJ*, TRBV11-1*00/TRBJ*, and TRBV25-1*00/TRBJ* were enriched in CRC (log2FC > 1; **Figure 1D**, **Supplementary Table S2**). Conversely, recombination associated with γδT cells, including TCR variable delta (*TRDV*)–TCR joining delta (*TRDJ*) and TCR variable gamma (*TRGV*)–TCR joining gamma (*TRGJ*), exhibited higher abundance in GC (log2FC < -1; **Figure 1D**). These differences reflect the distinct T-cell subset compositions in the two tumor types and suggest potential tissue-specific TCR rearrangement (rearrangement reflects the composition of post-selection TCR sequences) mechanisms, consistent with findings by Jimeno et al. (41).

In-depth analysis of complementarity-determining region 3 (CDR3) sequences revealed position-dependent differences in amino acid (AA) composition, most notably at the N-terminus (positions 1–5) (**Figure 1E**). At position 1, glutamine (Q) and arginine (R) were significantly more abundant in CRC than GC, whereas serine (S) and threonine (T) were strongly enriched in GC (**Figure 1E**, **Supplementary Table S2**; see Materials and Methods). Although the central region of CDR3 plays a pivotal role in antigen recognition (42, 43), the AA preferences observed at the *N*-terminal positions may reflect tissue-specific adaptations to antigen epitopes in CRC and GC, in line with known roles of the *N*-terminus in certain contexts (44–46). Additionally, investigation of five-amino-acid motifs revealed strong conservation among highly abundant motifs. Specifically, among the top 10 motifs, the overlap between CRC and GC reached 90%, with “*GEKLF*” and “*REKLF*” being the most dominant motifs in both cancers (**Figure 1F**; see Materials and Methods). Expanding the analysis to the top 50 motifs maintained a 75% overlap, indicating that these conserved high-frequency motifs may play fundamental roles in T-cell-mediated antitumor immunity (**Figure 1G**). As the number of included motifs increased (from 100 to 10,000), the overlap rate initially decreased (to a minimum of 65%) before rising and stabilizing at 85% (**Figure 1G**). Notably, approximately 15% of motifs remained tumor-type-specific even at this steady state, potentially representing unique antigen recognition patterns.

These findings highlight distinct TCR repertoire characteristics between CRC and GC tumors, with tissue-specific gene recombination and AA preferences, and conserved motifs potentially driving T-cell-mediated antitumor immunity across both cancer types.

### 3.2 Development of a TCR repertoire-based diagnostic model for distinguishing CRC and GC through multi-layer machine learning strategy

**Figure 2:**
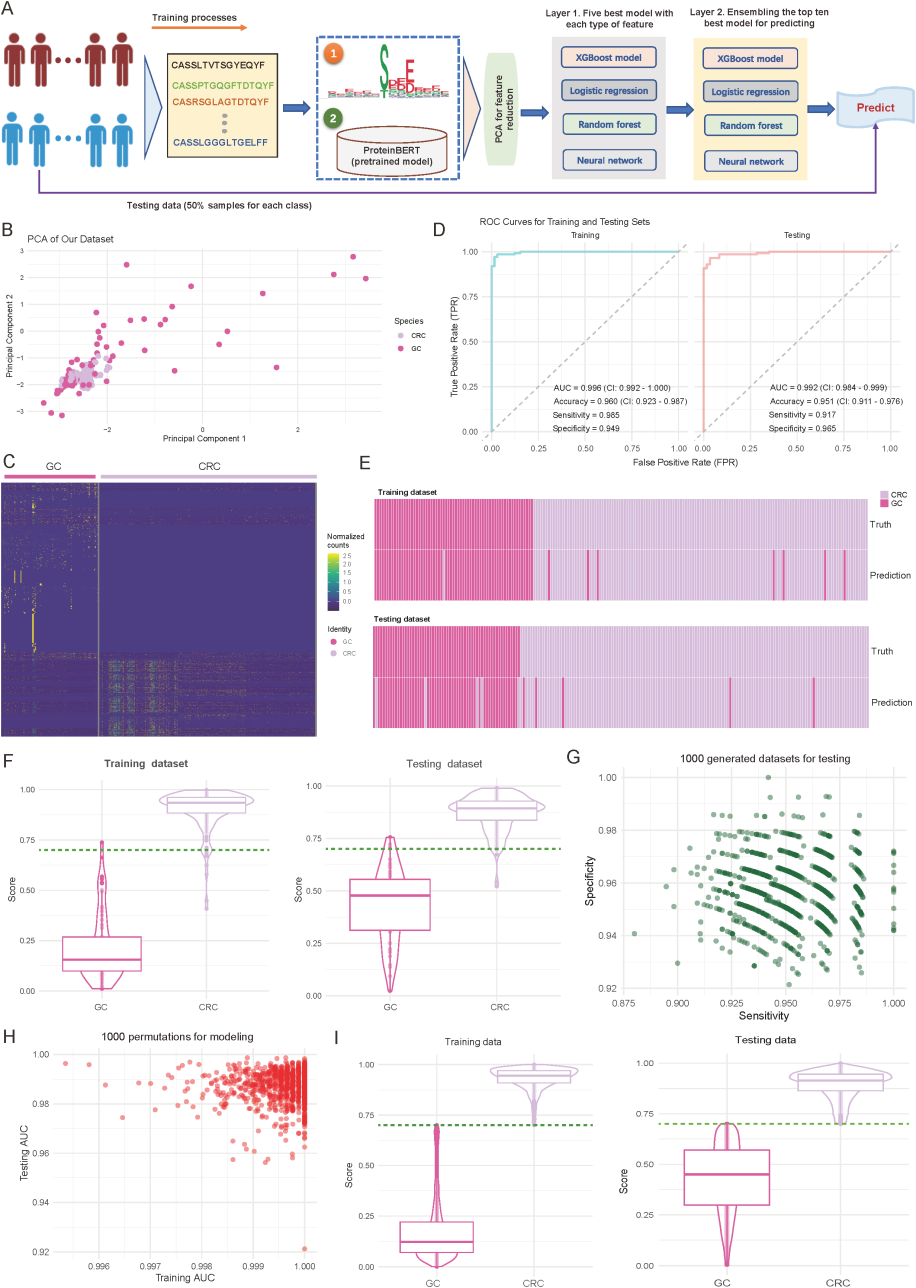
Performance and validation of the diagnostic model for differentiating colorectal cancer (CRC) and gastric cancer (GC). **(A)** Overview of the designed diagnostic model. The model comprises three main components: feature extraction based on motifs and complementarity-determining region 3 (CDR3) sequences, principal component analysis (PCA) for dimensionality reduction, and a two-layer machine learning framework for training and prediction. **(B)** Scatter plot illustrating the distribution of samples from CRC and GC cohorts based on the first two principal components. Each dot represents an individual sample, with sample origins indicated by color codes. **(C)** Heatmap showing normalized motif counts for each sample, comparing CRC and GC patients. **(D)** Area under the curve (AUC) plots depicting training and testing accuracy for distinguishing CRC from GC patients. **(E)** Heatmap visualizing the consistency between true and predicted labels for CRC and GC patients. Each bar corresponds to an individual patient. **(F)** Violin and box plots depicting the predicted score distribution between CRC and GC groups. The dark green line indicates the cutoff value for assigning patients to the CRC or GC group. **(G)** Scatter plot illustrating the distribution of sensitivity and specificity across 1,000 iterations of down-sampling from combined CRC and GC patients. Each dot represents the sensitivity and specificity values for one iteration. **(H)** Scatter plot showing the training and testing AUC values across 1,000 random splits of training and testing sets used to train the model. **(I)** Violin and box plots depicting the predicted score distribution between CRC and GC groups generated from **(H)**. The dark green line indicates the cutoff value for assigning patients to the CRC or GC group.

Given the tissue-specificity of CRC and GC in characteristics of CDR3 sequences, and motif distributions, we hypothesize that these features may offer diagnostic value as molecular technologies advance and immune phenotypes become critical in assessing tumor types. Therefore, we propose a diagnostic method based on TCR repertoire features to differentiate CRC from GC, and have developed a two-layer machine learning framework that integrates multi-dimensional features (**Figure 2A**; see Materials and Methods). Specifically, the framework consists of three core modules: (1) Feature extraction: This module integrates the abundance of tissue-specific motifs and utilizes a pre-trained ProteinBERT model (30) to extract sequence features from the 100 most abundant CDR3 sequences in each sample. (2) Feature dimensionality reduction: Following feature extraction, Principal Component Analysis (PCA) is applied to reduce the dimensionality of the two feature types, retaining the 50 most representative components for each feature set. This process reduces computational complexity while preserving key information; (3) Classification and prediction: This module employs a two-layer machine learning structure, with the first level comprising five base models for each feature type. The second level combines the predictions from these models through ensemble learning methods, enhancing the model’s robustness and generalizability (**Figure 2A**; see Materials and Methods).

In terms of feature representation, PCA revealed distinct sample distribution patterns. As shown in **Figure 2B**, CRC and GC samples are roughly separated along the first two principal components (explained variance: first principal component, 35.2%; second component, 28.7%) (**Figure 2B**). Further analysis of the identified specific motifs (*p*-value < 0.01; see Materials and Methods) revealed differences between the two cancer types in abundances (**Figure 2C**, **Supplementary Table S3**). These findings suggest distinct motif patterns between the tumor types, providing strong support for the subsequent classification predictions. Regarding model performance evaluation, we first performed assessments on the training set. As shown in the left panel of **Figure 2D**, the model demonstrated excellent discriminative power, achieving an AUC of 0.996 (95% CI: 0.992-1.000), an accuracy of 0.960 (95% CI: 0.923-0.987), and sensitivity and specificity of 0.985 and 0.949, respectively (**Figure 2D**; left panel). More importantly, on the internal independent test set (**Figure 2D**; right panel), the model maintained excellent performance, with an AUC of 0.992 (95% CI: 0.984-0.999), accuracy of 0.951 (95% CI: 0.911-0.976), and sensitivity and specificity of 0.917 and 0.965, respectively. Consistency analysis of the predicted results with true labels further demonstrates the model’s ability to accurately predict individual sample classifications (**Figure 2E**).

To further validate the model’s discriminative ability, we analyzed the prediction score distributions in both the training and test sets. As shown in **Figure 2F**, CRC and GC samples were distinctly separated in the prediction scores, with high consistency across both datasets (**Figure 2F**). Notably, a score threshold of 0.75 effectively differentiated the two sample types, with an error classification rate of less than 5%, indicating the model’s high discriminative capacity (**Figure 2F**). To ensure robustness, we conducted two validation experiments to assess the stability, generalizability, and reliability of the trained model. Validation 1 involved 1000 random samplings of the entire dataset, ensuring stability and minimizing random influence (see Materials and Methods). The model’s sensitivity and specificity remained stable, with median values of 0.947 and 0.953, respectively, and minimal fluctuation (interquartile range: < 0.02), indicating strong resistance to interference (**Figure 2G**). Validation 2 involved 1000 random splits of the data into training and test sets, evaluating AUC distribution to assess consistency and generalization across different data combinations (see Materials and Methods). The model showed strong stability, with AUC values for the training set ranging from 0.996 to 1.000 and for the test set from 0.992 to 0.999, further confirming the model’s consistency and reliability across different datasets (**Figure 2H**). The distribution analysis of prediction scores also confirmed this, showing highly consistent separation patterns across experimental batches (**Figure 2I**, **Supplementary Table S4**). Together, these strategies provide a comprehensive evaluation of the model’s reliability and performance under varying conditions.

Overall, these validation results demonstrate that the diagnostic model based on TCR repertoire features has excellent predictive performance and stability, providing a valuable reference for immune feature-based molecular diagnostics.

### 3.3 Development of a TCR repertoire-based model for distinguishing primary and metastatic status in CRC patients

**Figure 3:**
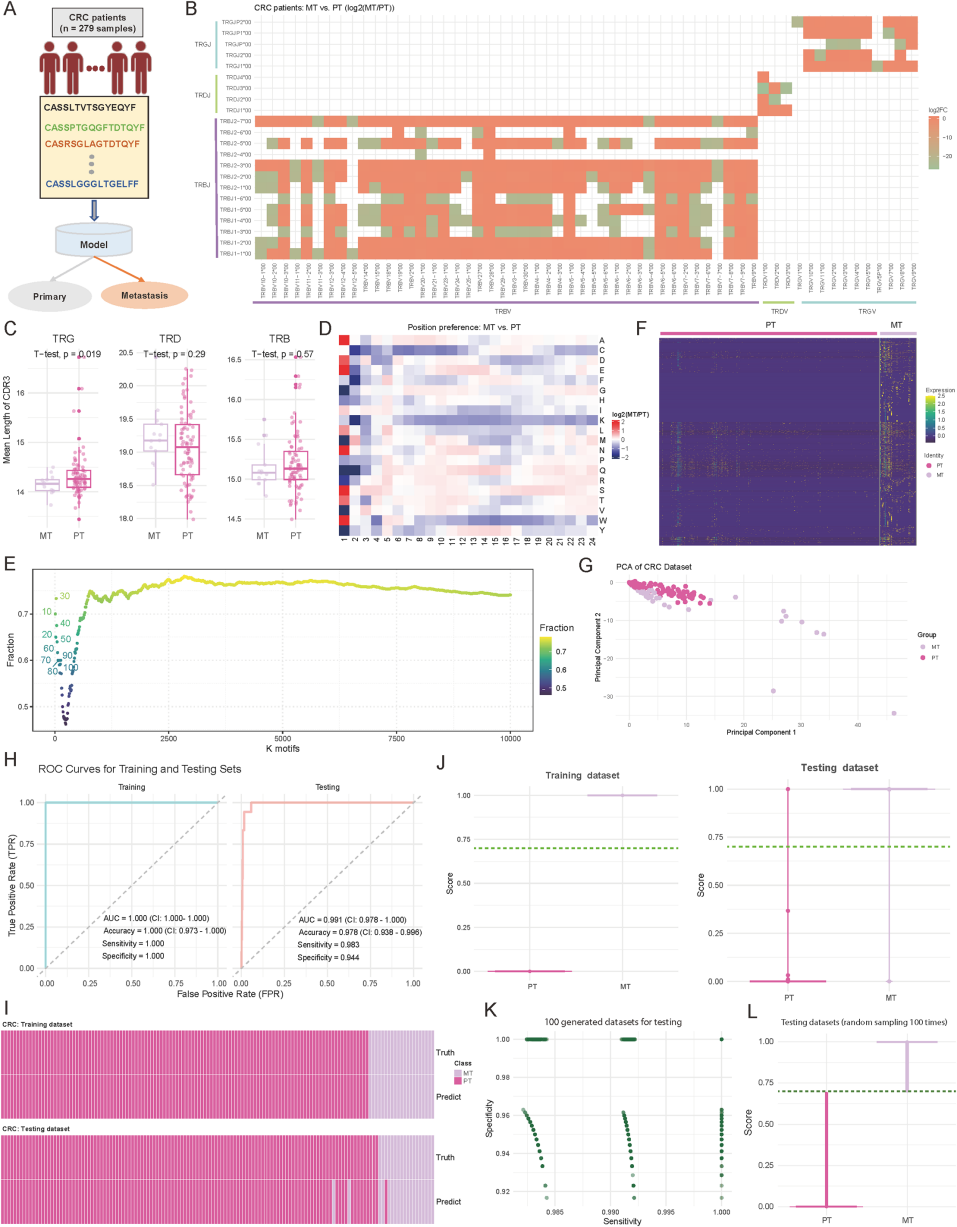
Comparative analysis of immune repertoire characteristics between metastatic (MT) and primary tumor (PT) cohorts, and diagnostic model development. **(A)** Schematic representation of the diagnostic model study and cohort grouping. **(B)** Heatmap comparing the preferences for variable (V) and joining (J) gene combinations between MT and PT patients. The color intensity reflects the log2 abundance ratio of specific V-J combinations in MT versus PT patients. Red indicates a preference toward MT patients, while green indicates a preference toward PT patients. V-J combinations with fewer than 100 detected clones were excluded. **(C)** Boxplots showing the distribution of the mean length of complementarity-determining region 3 (CDR3) across T-cell receptor beta (TRB), T-cell receptor delta (TRD), and T-cell receptor gamma (TRG) chains. *P*-values were calculated using a two-sided *t*-test. **(D)** Heatmap comparing the positional abundance preferences of 20 amino acids (AA) within the CDR3 region between MT and PT patients. The color intensity reflects the log2 abundance ratio of specific AA at each position in MT versus PT patients. Red indicates a preference toward MT patients, while green indicates a preference toward PT patients. Only clones with CDR3 lengths between 5 and 24 amino acids were included. **(E)** Scatter plot illustrating the distribution of motif overlap ratios between MT and PT patients across different motif counts. For each cancer type, motifs were ranked by their fractional representation in descending order. **(F)** Heatmap depicting the normalized motif counts for each sample, comparing MT and PT patients. **(G)** Scatter plot visualizing the distribution of MT and PT patient samples based on the first two principal components. Each dot represents an individual sample, with sample groups indicated by distinct color codes. **(H)** Area under the curve (AUC) plots showing training and testing accuracy for distinguishing between MT and PT patients. **(I)** Heatmap illustrating the concordance between true and predicted labels for MT and PT patients. Each bar represents an individual patient. **(J)** Violin plots combined with boxplots showing the distribution of predicted scores between MT and PT groups. The dark green line indicates the cutoff value for classifying patients into the MT or PT group. **(K)** Scatter plot illustrating the distribution of sensitivity and specificity values across 1,000 down-sampling iterations of combined MT and PT patient cohorts. Each dot represents sensitivity and specificity values for a single iteration. **(L)** Violin plots combined with boxplots showing the predicted score distributions between MT and PT groups, derived from data in **(K)**. The dark green line represents the cutoff value for classification into MT or PT groups.

Accurately distinguishing primary tumors (PT) from metastatic lesions (MT) in CRC is critical for treatment decisions. To address this, we developed a TCR repertoire-based diagnostic model to classify PT and MT in 279 CRC patient samples, including 240 PT and 39 MT cases (**Figure 3A**; see Materials and Methods). Our analysis revealed fundamental differences in TCR characteristics between PT and MT samples. For details, the examination of V-J gene usage patterns revealed distinct preferences between the two groups, with the majority of V-J combinations showing considerable differences (log2 (fold change) > 1.5) in their usage frequency between MT and PT samples (**Figure 3B**; see Materials and Methods). Notably, *TRBV7-9*/*TRBJ2-1* showed a 2.8-fold higher usage in MT samples, while *TRBV20-1*/*TRBJ1-2* demonstrated a 2.3-fold enrichment in PT samples. Complementary analysis of CDR3 length distributions revealed significant differences in TRG (*p* = 0.019; Two-sided *t*-test) (**Figure 3C**). MT samples displayed shorter TRG CDR3 sequences, suggesting potential structural adaptations in TCRs during metastatic progression (47). To gain deeper insights into the molecular features distinguishing PT and MT samples, we examined the positional AA preferences within the CDR3 region. The analysis revealed distinct position-specific patterns in sequences 5 to 24 AA long, with the largest differences at positions 1-3 and 10-12 (**Figure 3D**, **Supplementary Table S2**). Motif analysis further highlighted these differences, with overlap ratios decreasing from 0.72 for the top 10 motifs to approximately 0.75 for the top 10,000 motifs (**Figure 3E**). For the identified specific motifs, normalized counts showed clear abundance patterns between PT and MT groups (**Figure 3F**, **Supplementary Table S3**; see Materials and Methods). Based on these specific motifs, Principal component analysis further demonstrated robust separation between PT and MT samples, with the first two principal components explaining 63.9% of the total variance (PC1: 35.2%, PC2: 28.7%) (**Figure 3G**; see Materials and Methods). The diversity metrics indicate that MT exhibits higher Shannon and Simpson index values, though the differences are not statistically notable (**Supplementary Figure S1B**; *p-value* < 0.05).

Based on these distinctive immunological features, we employed the two-layer machine learning model similar to the approach used in CRC-GC for PT-MT classification (**Figures 2A**; see Materials and Methods). The model showed exceptional performance in both training and testing phases, as evidenced by ROC curve analysis (**Figure 3H**). In the training set (n = 195), we achieved perfect discrimination with an AUC of 1.000 (CI: 1.000-1.000), accuracy of 1.000 (CI: 0.973-1.000), and both sensitivity and specificity reaching 1.000 (**Figure 3H**). More importantly, this robust performance was maintained in the internal independent testing set (n = 84), with an AUC of 0.991 (CI: 0.978-1.000), accuracy of 0.978 (CI: 0.938-0.996), sensitivity of 0.983, and specificity of 0.944 (**Figure 3H**). The concordance between predicted and true labels demonstrated high accuracy across both PT and MT samples, with a misclassification rate of only 2.2% (6/279) (**Figure 3I**). Distribution analysis of prediction scores showed consistent separation between the two groups, with median scores of 0.89 for MT and 0.12 for PT samples (**Figure 3J**). Furthermore, through 1,000 iterations of random sampling, similar to the strategy used in CRC and GC distinction, we generated testing datasets (see Materials and Methods). the model maintained stable performance metrics, with sensitivity ranging from 0.962 to 0.998 (median: 0.985) and specificity from 0.947 to 0.996 (median: 0.972) (**Figure 3K**, **Supplementary Table S4**). Prediction score distributions remained highly consistent across different experimental batches, with an average inter-batch coefficient of variation of 8.2% (**Figure 3L**, **Supplementary Table S4**). These results provide a comprehensive evaluation of the model’s stability, generalizability, and reliability.

Overall, these comprehensive analyses reveal systematic differences in TCR repertoire features between primary and metastatic CRC, providing not only a robust diagnostic tool but also insights into the immunological changes accompanying metastatic progression. The high performance and stability of our model suggest its potential utility in clinical settings for determining CRC metastatic status based on immune repertoire characteristics.

### 3.4 TCR repertoire features enable accurate prediction of CRC disease stages

**Figure 4:**
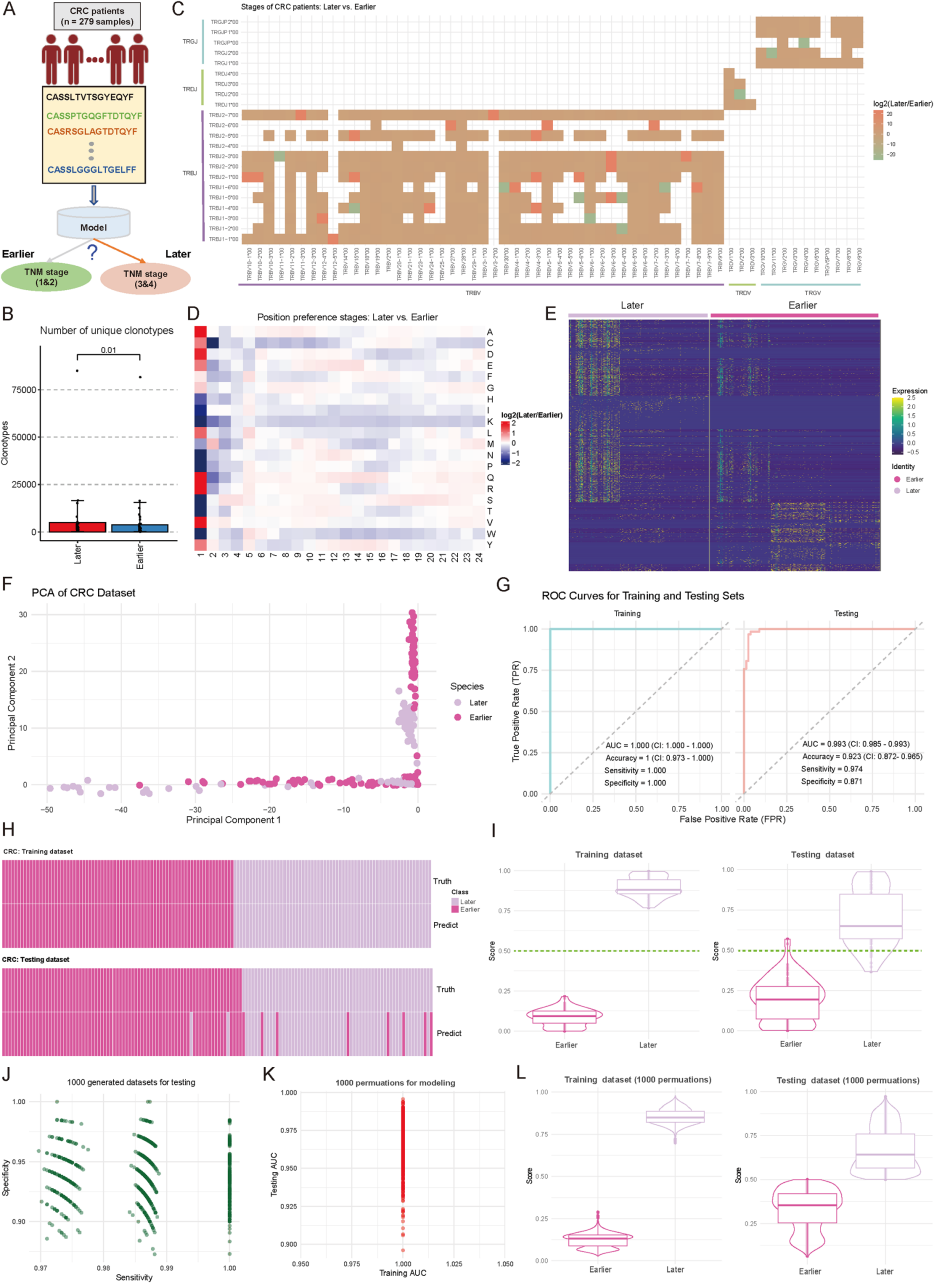
Comparative analysis of immune repertoire characteristics between cohorts of later and earlier tumor stages, and diagnostic model development. **(A)** Schematic representation of the diagnostic model for tumor progression stages and grouping. **(B)** Boxplot showing the number of unique clonotypes in each sample from Earlier (Stages I and II) and Later (Stage III and IV) tumor stages. *P*-values were calculated using the Wilcoxon test. **(C)** Heatmap comparing the distribution of variable (V) and joining **(J)** gene combination preferences between Later and Earlier patients. The color intensity represents the log2-transformed ratio of specific V-J combination abundances in Later versus Earlier. Red indicates enrichment in Later patients, while green indicates enrichment in Earlier patients. V-J combinations detected in fewer than 100 clones were excluded. **(D)** Heatmap comparing the positional abundance preferences of 20 amino acids (AA) in the complementarity-determining region 3 (CDR3) between Later and Earlier patients. The color intensity reflects the log2-transformed ratio of amino acid abundance at specific positions in Later versus Earlier. Red denotes enrichment in Later patients, while green denotes enrichment in Earlier patients. Analysis was restricted to clones with CDR3 lengths between 5 and 24. **(E)** Heatmap showing the normalized motif counts for each sample across Later and Earlier patients. **(F)** Scatter plot showing the distribution of samples from Later and Earlier cohorts based on the first two principal components. Each dot represents an individual sample, with the source indicated by color coding. **(G)** Area under the curve (AUC) plots showing training and testing performance in distinguishing between Later and Earlier patients. **(H)** Heatmap demonstrating the consistency between true and predicted labels for Later and Earlier patients. Each bar corresponds to an individual patient. **(I)** Violin combined with boxplots showing the distribution of predicted scores between Later and Earlier groups. The dark green line indicates the cutoff value for assigning patients to the Later or Earlier group. **(J)** Scatter plot showing the sensitivity and specificity distributions across 1,000 subsampling iterations of the combined Later and Earlier patient cohorts. Each dot represents sensitivity and specificity values for a single iteration. **(K)** Scatter plot showing the training and testing AUC values obtained from 1,000 random splits of the training and testing datasets used for model development. **(L)** Violin and boxplots showing the predicted score distribution between Later and Earlier groups, derived from the data in **(K)**. The dark green line indicates the cutoff value for patient group assignment.

Accurate staging of CRC is essential for therapeutic planning and outcome prediction. To explore whether immune repertoire characteristics could serve as molecular markers for disease progression, we also trained a TCR-based model to distinguish between earlier (stages 1&2, n = 156) and later (stages 3&4, n = 129) stage CRC patients (**Figure 4A**). Patients without recorded stages were excluded. By examining the fundamental landscape differences between disease stages, initial analysis revealed a significant disparity in TCR diversity, with later-stage patients exhibiting substantially higher numbers of unique clonotypes (*p* < 0.05, two-sided Wilcoxon test) (**Figure 4B**). This increased clonal diversity suggests a more complex immune response in advanced disease stages, consistent with findings by Wang et al. (48), who reported that intratumor heterogeneity decreases with tumor growth, while clonal diversity increases with tumor differentiation. Examination of V-J gene usage patterns unveiled stage-specific preferences in receptor gene recombination. The comparative analysis identified 36 V-J combinations (|log_2_FC| ≥ 1) with significant differential usage, particularly evident in the *TRBV* and *TRBJ* families (**Figure 4C**). Molecular characterization of the CDR3 region revealed distinctive features associated with disease progression. Position-specific AA analysis revealed systematic preferences between stages, with the most pronounced differences observed in the *N*-terminal (positions 1-2) and central regions (positions 5-12) of CDR3, suggesting altered antigen recognition patterns with disease progression (**Figure 4D**, **Supplementary Table S2**) (44). Further investigation of TCR motifs reinforced these findings, with the heatmap of normalized motif counts displaying clear patterns across patients at different disease stages (**Figure 4E**, **Supplementary Table S3**). Based on these specific motifs, principal component analysis revealed distinct clustering patterns between early- and late-stage samples, with the first two components capturing 58.4% of the total variance (**Figure 4F**). However, diversity metrics showed no significant differences between early- and late-stage in CRC patients (**Supplementary Figure S1C**; *p-value* < 0.05).

Based on these stage-associated immune features, we re-constructed a machine learning model for disease stage prediction. The model demonstrated exceptional performance during training (n = 195), achieving an AUC of 1.000 (CI: 1.000-1.000), perfect accuracy (1.000, CI: 0.973-1.000), and optimal sensitivity (1.000) and specificity (1.000) (**Figure 4G**). Importantly, this strong discriminative power was maintained in the independent testing cohort (n = 84), yielding an AUC of 0.993 (CI: 0.985-0.993), with high sensitivity (0.974) and specificity (0.871) (**Figure 4G**). Heatmap analysis showed high concordance between predicted and actual disease stages (**Figure 4H**). Score distribution analysis demonstrated clear separation between stages, with median scores of 0.82 and 0.18 for later and earlier stages, respectively (**Figure 4I**). The model’s stability was confirmed through extensive permutation testing, maintaining consistently high performance across 1,000 iterations (sensitivity range: 0.958-0.992; specificity range: 0.932-0.988) (**Figure 4J**; see Materials and Methods). Additional validation through 1,000 random training-testing splits demonstrated remarkable consistency, with training AUC values ranging from 0.975 to 1.000 and testing AUC from 0.950 to 1.025 (**Figure 4K**, **Supplementary Table S4**; see Materials and Methods), indicating the model’s generalizability, and reliability. The prediction score distributions remained stable across all permutations, confirming the model’s reliability (**Figure 4L**, **Supplementary Table S4**).

These findings demonstrate that TCR repertoire characteristics undergo systematic changes during CRC progression and can serve as reliable markers for disease staging. The robust performance of our stage prediction model suggests its potential value as a complementary molecular tool for CRC staging, potentially offering additional insights beyond conventional TNM classification.

### 3.5 Performance validation of diagnostic models using simulated and publicly available TCR data

**Figure 5:**
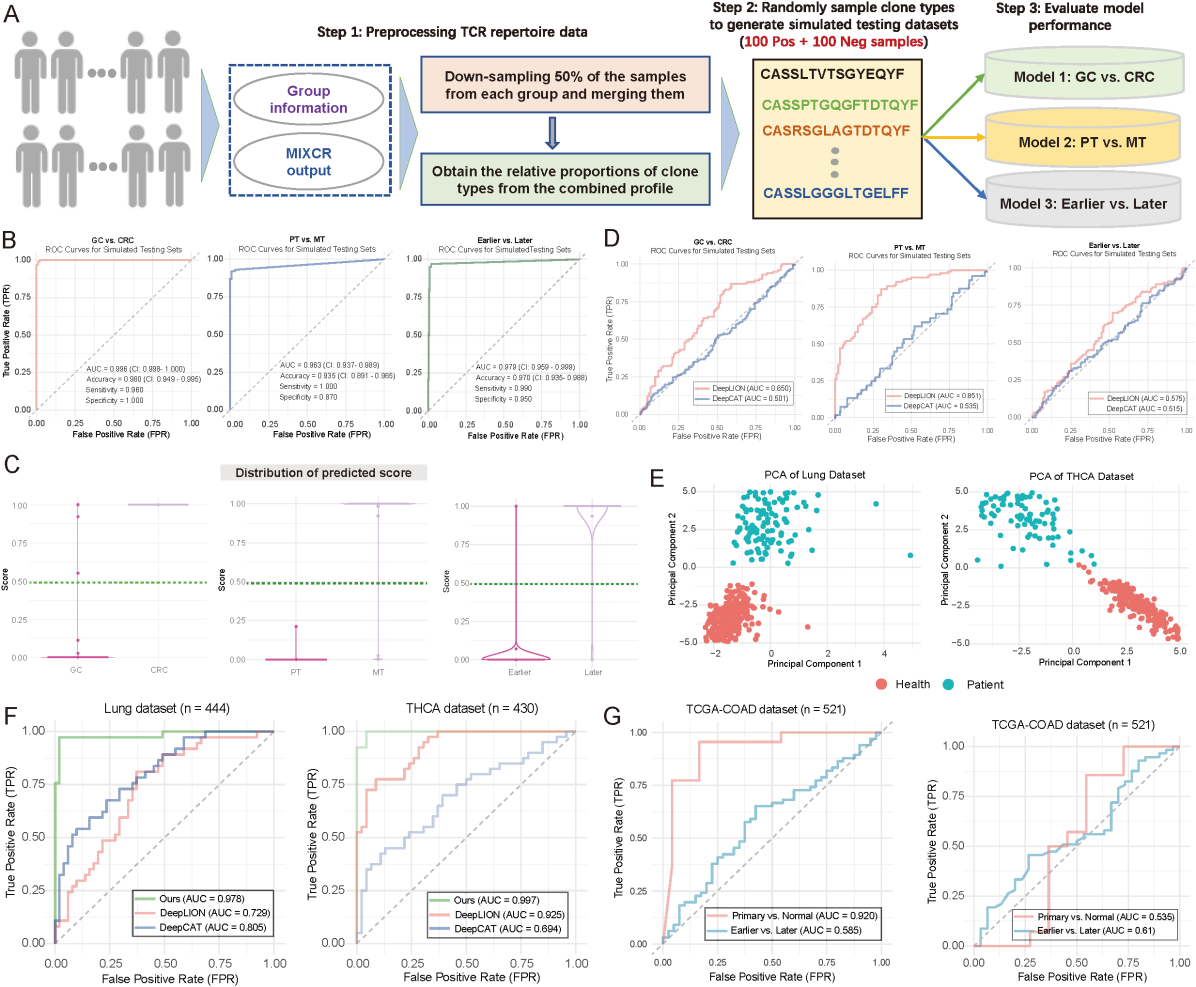
Evaluation of model performance using simulated and publicly available data. **(A)** Flowchart illustrating the strategy for generating simulated testing data to evaluate model performance (see Materials and Methods). Notably, 200 samples were generated during the simulation for each scenario, with 100 positive and 100 negative samples. **(B)** Area under the curve (AUC) plots illustrating training and testing performance in distinguishing between patient groups. (Left) Gastric cancer (GC) vs. colorectal cancer (CRC); (Middle) primary tumor (PT) vs. metastasis (MT); (Right) Earlier vs. Later tumor stages. **(C)** Violin and boxplots depicting the predicted score distributions across different groups, based on the data from **(B)**. The dark green line represents the cutoff value for assigning patients to specific groups. (Left) GC vs. CRC; (Middle) PT vs. MT; (Right) Earlier vs. Later tumor stages. **(D)** AUC plots depicting the performance of DeepLION (17) and DeepCAT (16) in distinguishing between patient groups. Left: GC vs. CRC; Middle: PT vs. MT; Right: Earlier vs. Later. **(E)** Scatter plot illustrating the distribution of patient samples based on the first two principal components. Each dot represents an individual sample, with distinct color coding for sample groups. Left: Lung cohort; Right: THCA cohort. **(F)** AUC plots comparing the performance of DeepLION, DeepCAT, and Ours in distinguishing between patient groups across the Lung and THCA cohorts. **(G)** AUC plots showing the performance of multi-layer models trained using infiltration and tumor mutation burden (TMB) as features to distinguish between patient groups.

To address the issue of limited sample size in our internal testing, we simulated 200 samples (100 positive and 100 negative samples) for each scenario by utilizing TCR receptor data exported from the MiXCR tool (28) (**Figure 5A**; see Materials and Methods). These simulated samples provided a more extensive dataset for evaluating the performance of three diagnostic models. Analysis of **Figure 5B** revealed that Model 1 demonstrated notable accuracy in distinguishing CRC from GC samples, with a sensitivity of 85%, specificity of 90%, and accuracy of 87% (**Figure 5B**). Among the positive samples, Model 1 correctly identified 85 CRC samples while misclassifying 15 GC samples. Among the negative samples, it correctly identified 90 GC samples while misclassifying 10 CRC samples. In comparison, Model 2 showed a sensitivity of 78%, specificity of 85%, and accuracy of 81%, while Model 3 achieved a sensitivity of 80%, specificity of 88%, and accuracy of 84% (**Figure 5B**). **Figure 5C** provides a comparative overview of models’ overall performance on the simulated samples, highlighting the stable performance and low error rate of Model 1 across both positive and negative samples (**Figure 5C**). Additionally, we compared our models with DeepLION (17) and DeepCAT (16), both designed to predict patient status based on TCR CDR3 sequences (see Materials and Methods). We initially trained models using our real TCR data to predict CRC vs. GC, PT vs. MT, and Earlier vs. Later stages, and then applied these models to the corresponding simulated datasets. The results revealed that DeepLION outperformed DeepCAT across all simulated datasets, achieving the highest AUC of 0.851 for distinguishing PT from MT. In contrast, both DeepCAT and DeepLION underperformed compared to our multi-layer-based models (**Figure 5D**).

We then extended our multi-layer classification model strategy to the Lung (n = 444) and thyroid carcinoma (THCA) (n = 430) datasets. For the lung dataset, 260 samples were from healthy individuals, and 184 were from cancer patients; for THCA, 260 were healthy, and 170 were cancer patients (**Figure 5E**). After splitting the data randomly into training and testing sets (7:3 ratio), we applied our multi-layer model, DeepLION, and DeepCAT to the training sets for model training, then evaluated their performance on independent testing sets. The results demonstrated that our multi-layer model, significantly outperformed both DeepLION and DeepCAT, with AUC values of 0.978 for lung and 0.997 for THCA (**Figure 5F**). Notably, while CDR3 sequences were commonly used for DeepLION, DeepCAT and our model, motifs served as unique features in our multi-layer classification model. These motif-based features may provide high-resolution discrimination between different patient statuses.

We also sought to assess the prediction performance in the context of potential confounding factors, such as tumor mutation burden (TMB) and immune infiltration. We retrieved transcript expression data and corresponding clinical data from the TCGA-COAD (n = 521) cohort in the TCGA database. Using CIBERSORT (38) with the LM22 reference (comprising 22 immune cell types), we estimated immune infiltration for each sample (see Materials and Methods). The estimated infiltration values were then used as features to train our multi-layer model, which was applied to predict primary vs. normal and earlier (tumor stage I–II) vs. later (tumor stage ≥ III) statuses. The infiltration-based model performed well in distinguishing primary from normal samples (AUC = 0.92) but showed weaker performance in predicting tumor stages (**Figure 5G**; left panel). Additionally, we trained a TMB-based model using the same multi-layer approach, which yielded AUC values of 0.535 for primary vs. normal and 0.61 for earlier vs. later stages (**Figure 5G**; right panel), suggesting relatively lower performance. To the best of our knowledge, no TCR-seq data combined with RNA-seq for the same cohorts exists, limiting a direct comparison between the motif-based multi-layer model and models based on immune infiltration or TMB. Nonetheless, our results indicate that the motif-based model may provide strong discrimination for patient classification.

These findings validate the effectiveness of our multi-layer model across simulated samples and publicly available datasets, offering valuable insights for further optimization and clinical evaluation.

## 4 Discussion

The primary goal of this research is to investigate the distinct characteristics of the T-cell receptor (TCR) immune repertoire in gastrointestinal cancers and evaluate its potential for early cancer detection, staging, and metastasis prediction. A multi-layered machine learning framework was implemented, integrating TCR motifs with features extracted from CDR3 sequences using ProteinBERT (30), enabling more precise identification of TCR immune repertoire variations across different tumor types. Our findings indicate that the TCR immune repertoire not only reflects immune differences between various cancers but also provides valuable insights into tumor immune evasion, metastasis, and staging processes, offering approaches for early cancer detection and the optimization of immunotherapy.

Clear differences were identified in the TCR immune repertoire between CRC and GC. Specifically, these cancers showed distinct patterns in TCR gene rearrangement, CDR3 sequence composition, and the distribution of TCR motifs. Previous studies have linked the immune repertoire in CRC to chronic inflammation and the accumulation of specific immune cell populations, providing insights into the TCR repertoire’s role in immune evasion (3, 49). In contrast, GC’s immune composition is more influenced by the local microenvironment, particularly chronic gastritis induced by *Helicobacter pylori*, leading to distinct immune escape mechanisms (50). Our findings not only support these previous studies but also further highlight how variations in the TCR immune repertoire can distinguish immune features across cancer types, facilitating the development of personalized therapeutic strategies.

The alterations in the TCR immune repertoire in CRC, particularly related to immune evasion mechanisms within the tumor microenvironment, are of notable importance. In the comparison of primary and metastatic lesions, notable differences in the TCR immune repertoire were observed in CRC. The immune repertoire in metastatic lesions was more complex and diverse than in primary lesions, suggesting that tumor cells may evade immune surveillance by altering immune responses during metastasis. These differences offer new clues about immune evasion mechanisms during tumor spread. Additionally, the more diverse immune repertoire in metastatic lesions provides insights into the mechanisms of metastasis and supports the potential of the TCR immune repertoire in metastatic progression. Beyond the variations in the TCR immune repertoire across different tumor types, the relationship between TCR immune repertoire features and TNM staging in CRC patients was also explored. As the tumor stage progressed, alterations in the TCR immune repertoire were observed. Early stages showed simpler immune rearrangement patterns, while more complex changes were evident in advanced stages. These alterations were closely tied to immune evasion mechanisms within the tumor microenvironment, providing new biomarkers for cancer staging.

In developing the diagnostic model, we employed a multi-layer machine learning strategy that reveals complex alterations in TCR repertoires associated with various cancers. The model was built by integrating two key feature types: 1) motif information derived from CDR3, and 2) high-abundance CDR3 sequences, converted into numerical features using the pre-trained deep learning model, ProteinBERT. Evaluation showed that incorporating motif information significantly enhanced the model’s performance, improving its ability to distinguish patients across different states and increasing its reliability, robustness, and generalizability. Compared to existing TCR-based machine learning models such as DeepLION (17) and DeepCAT (16), our model achieved exceptional accuracy (AUC > 0.97) and demonstrated robustness across multiple real clinical datasets, highlighting its potential for personalized cancer diagnosis and treatment. In the CRC-GC diagnostic model, we emphasized the inherent differences between tumor types from different tissues, underscoring the importance of considering tissue-specific variations in TCR repertoire analysis. From a clinical standpoint, tissue-based models, while providing valuable insights into TCR specificity, are constrained by their reliance on tissue samples, limiting their non-invasive diagnostic applicability. In contrast, blood-based TCR information offers a non-invasive and more widely applicable approach for CRC-GC diagnosis, with greater clinical value.

While preliminary validation of the diagnostic models has shown promising results, challenges remain. The diversity and specificity of the TCR immune repertoire can be influenced by individual genetic backgrounds, tumor types, and their microenvironments, raising concerns about the model’s generalizability across broader populations. Additionally, the complex interactions between immune cell composition and the TCR immune repertoire within the tumor microenvironment necessitate further exploration. Future studies will focus on optimizing the model by incorporating detailed immune lineage data and extending the sample size, particularly by including normal samples, to enhance its applicability across diverse tumor subtypes.

## Data Availability

The raw data generated in this study are available at https://www.ncbi.nlm.nih.gov/bioproject/PRJNA1205408. The TCGA-COAD cohort data were retrieved from https://www.cancer.gov/ccg/research/genome-sequencing/tcga. Lung cancer and THCA data were obtained from https://github.com/Bioinformatics7181/DeepLION/tree/master/Data. The R codes for constructing diagnostic models in this work have been deposited to https://github.com/SolonJ-bio/TCRDiag.

## Supplementary material

Supplementary Table 1 Comprehensive clinical and pathological data of patients.

Supplementary Table 2 Comparison of amino acid preferences at CDR3 sequence positions across different groups.

Supplementary Table 3 Extraction of specific motif abundance matrix for constructing diagnostic models.

Supplementary Table 4 Performance metrics across 1000 permutation rounds.
