# Supplementary material for: T-cell receptor dynamics in digestive system cancers: a multi-layer machine learning approach for tumor diagnosis and staging
Source: Front Immunol. 2025 Apr 8;16:1556165. doi: 10.3389/fimmu.2025.1556165 (PMC12011560; doi:10.3389/fimmu.2025.1556165)
Supplement: Supplementary file 1 [file DataSheet1.zip › Supp.Figures.pdf]

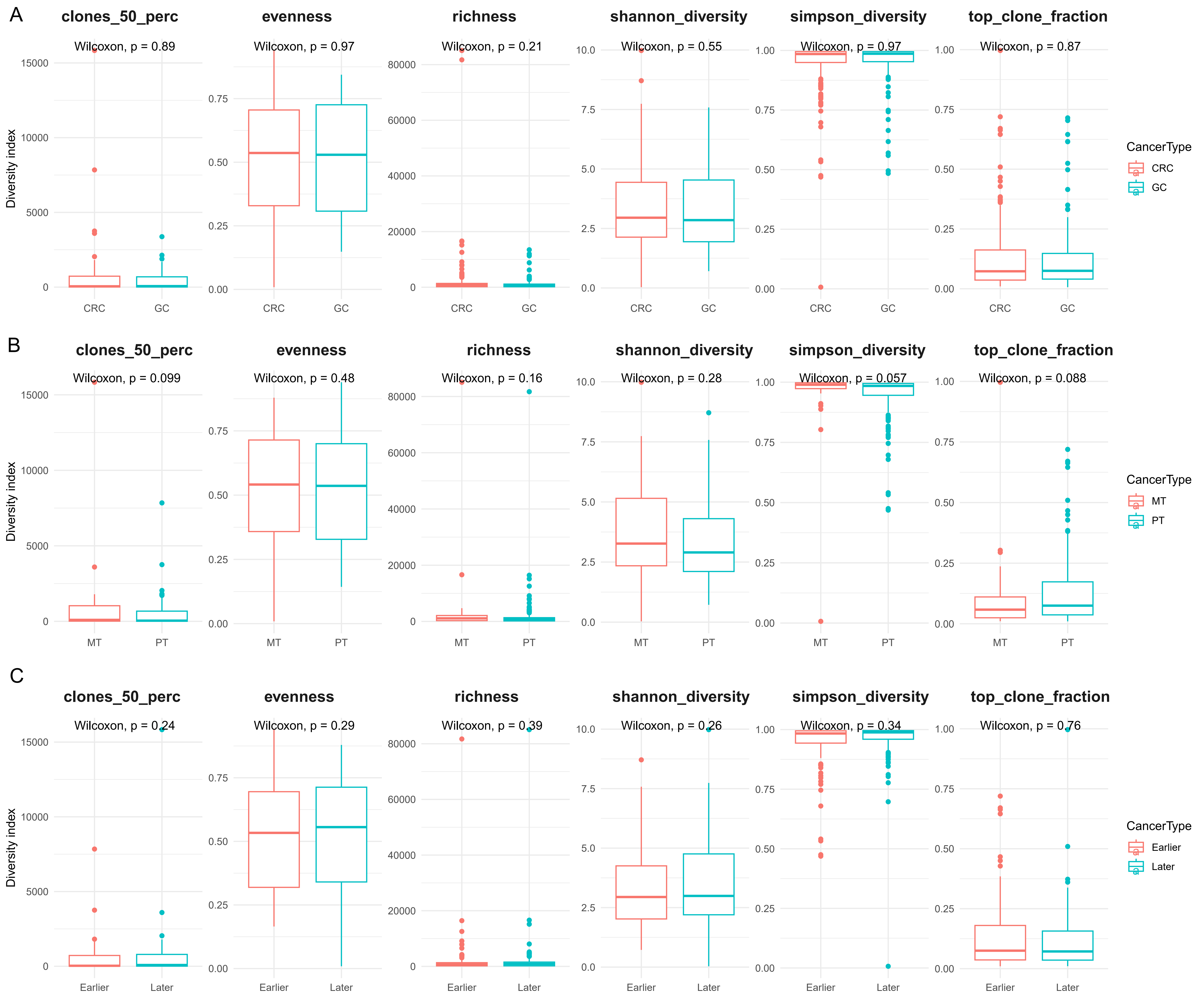
A
clones_50_perc
Wilcoxon, p = 0.89
evenness
Wilcoxon, p = 0.97
richness
Wilcoxon, p = 0.21
shannon_diversity
Wilcoxon, p = 0.55
simpson_diversity
Wilcoxon, p = 0.97
top_clone_fraction
Wilcoxon, p = 0.87
Diversity index
CRC
GC
CancerType
CRC
GC
B
clones_50_perc
Wilcoxon, p = 0.099
evenness
Wilcoxon, p = 0.48
richness
Wilcoxon, p = 0.16
shannon_diversity
Wilcoxon, p = 0.28
simpson_diversity
Wilcoxon, p = 0.057
top_clone_fraction
Wilcoxon, p = 0.088
Diversity index
MT
PT
CancerType
MT
PT
C
clones_50_perc
Wilcoxon, p = 0.24
evenness
Wilcoxon, p = 0.29
richness
Wilcoxon, p = 0.39
shannon_diversity
Wilcoxon, p = 0.26
simpson_diversity
Wilcoxon, p = 0.34
top_clone_fraction
Wilcoxon, p = 0.76
Diversity index
Earlier
Later
CancerType
Earlier
Later

Figure S1. Diversity metrics of T-cell receptors across patient groups. Shannon diversity, Simpson diversity, richness, evenness, top clone fraction, and the number of clones contributing to 50% of the repertoire were obtained. Shannon diversity was calculated as $-\sum \boldsymbol{p_i} \mathbf{log}\, \boldsymbol{p_i}$, where $\boldsymbol{p_i}$ represents the proportion of each clone. Simpson diversity was estimated as $\mathbf{1} - \sum \boldsymbol{p_i} \mathbf{log}\, \boldsymbol{p_i}$. Richness was defined as the total number of unique clones, and evenness was derived as the ratio of Shannon diversity to the logarithm of richness. The top clone fraction was determined as the maximum clone proportion, and the number of clones constituting 50% of the total repertoire was obtained by summing the largest clone proportions until the cumulative sum exceeded 0.5. p-values were obtained using two-sided Wilcoxon test.
